# H-Type Hypertension Is a Risk Factor for Cerebral Small-Vessel Disease

**DOI:** 10.1155/2020/6498903

**Published:** 2020-02-07

**Authors:** Tan Li, Xueyun Liu, Shanshan Diao, Yan Kong, Xiaoyu Duan, Si Yang, Sanjiao Liu, Qi Fang, Xiuying Cai

**Affiliations:** Department of Neurology, The First Affiliated Hospital of Soochow University, No. 899 Pinghai Road, Suzhou, Jiangsu 215006, China

## Abstract

**Background:**

The correlation between H-type hypertension and cerebral small-vessel diseases (CSVD) remains uncertain.

**Objective:**

The present study was designed to explore the possible relationship between H-type hypertension and CSVD spectrum and total burden.

**Method:**

We included 329 patients in the present study and divided them into four groups: the H-type hypertension group, isolated hypertension group, isolated hyperhomocysteinemia (HHcy) group, and control group. Clinical variables of interest and the MR examination sequences were obtained. We counted the presence of each CSVD feature and rated the total burden of CSVD on an ordinal scale from 0 to 4 according to a recent described score rule.

**Result:**

The results showed that H-type hypertension was associated with the presence of cerebral microbleeds (CMBs), and the severity of white-matter hyperintensities (WMHs) and peripheral vascular space (PVS). CSVD total burden was significantly related to age (OR: 1.059, 95% CI: 1.037–1.082), systolic pressure (OR: 1.122, 95% CI: 1.007–1.136), triglycerides (OR: 1.386, 95% CI: 1.037–1.854), isolated HHcy (OR: 4.154, 95% CI 1.836–9.401), and H-type hypertension (OR: 5.028, 95% CI: 2.323–10.883). Also, we further observed hypertension and HHcy had a synergistic effect on CSVD total burden (OR: 2.776, 95% CI: 1.564–4.927).

**Conclusion:**

H-type hypertension was associated with CSVD total burden and CSVD spectrum, which deserves further prevention measures. Furthermore, hypertension and HHcy had a synergistic effect on CSVD total burden.

## 1. Introduction

Cerebral small-vessel disease (CSVD) is an intrinsic disorder of small arteries, arterioles, venules, and capillaries of the brain [1]. From a clinical point of view, CSVD contributes to a risk of cognitive decline, dementia, and stroke and causes considerable worsening of cognitive function, gait, and balance [2]. In recent years, the development of neuroimaging has improved the diagnostic rate of CSVD. The recognized neuroimaging spectrum ascribable to CSVD has been expanded, including leukoaraiosis, cerebral microbleeds, lacunar infarcts, perivascular spaces, and brain atrophy currently [1]. Magnetic resonance (MR) is the gold standard imaging for CSVD, and four closely correlated features are markers on brain MR: white-matter hyperintensities (WMHs), lacunes, cerebral microbleeds (CMBs), and perivascular space (PVS).

Recently, a total small-vessel disease (SVD) burden score has been proposed [3–5], which captures the global effect of cerebral SVD and quantifies the global burden with a combined score. In this score, one point is allocated to each of the following: presence of lacunes, presence of microbleeds, moderate-to-severe WMHs, and PVS grated. The score has been tested partly for association with vascular risk factors or stroke subtype in a few studies [6, 7].

H-type hypertension, which refers to the concurrence of primary hypertension and elevated homocysteine levels, is a special hypertension type. In China, approximately 75% of the hypertensive patients simultaneously have hyperhomocysteinemia (HHcy). Previous studies suggested that H-type hypertension could be a significant risk factor for cardiocerebrovascular disease and that their effects were synergistic [8, 9]. Thus, H-type hypertension has received increasing attention over the years and has become a hot-spot. However, few studies have assessed the association between H-type hypertension and CSVD.

This retrospective study was designed to investigate the impact of H-type hypertension on CSVD spectrum and CSVD total burden. Also, we aimed to screen the risk factors of CSVD and prevent CSVD at an early stage.

## 2. Methods and Materials

### 2.1. Study Design and Subjects

The present study was performed in Stroke Center of First Hospital Affiliated to Soochow University and included 329 patients diagnosed with ischemic stroke who were admitted to our hospital from October 2015 to February 2018. The patients who underwent admission and finished MR-based imaging were included. The participants were divided into four groups: the control group (patients with neither hypertension nor HHcy), the isolated hypertension group, the isolated HHcy group, and the H-type hypertension group. At least 2 trained neurologists from our stroke center evaluated the clinical features and diagnostic test results. All data were analyzed anonymously. Ethical approval for this study was obtained from the ethics committees of the First Hospital Affiliated to Soochow University, and informed consent was obtained from all of our participants.

### 2.2. Clinical Information

Clinical variables of interest included age (calculated according to the ID birth date), gender, education level, and marital status. Lifestyle factors including smoking and alcohol consumption, past medical history, family history, disease history of hypertension history, diabetes history, stroke history, hyperlipidemia history, and coronary heart disease history were obtained. Hypertension was defined as the presence of any of the following: systolic blood pressure ≥140 mmHg or diastolic pressure ≥90 mmHg twice in quiet conditions or having self-reported history of hypertension. Diabetes mellitus was defined as the presence any of the following: fasting serum glucose >7.0 mmol/L or postprandial 2 h plasma glucose >11.1 mmol/L or having previous history of diabetes. Hyperlipidemia was defined as having elevated level of one of triglyceride, total cholesterol, or low density lipoprotein. Venous blood samples from the participants were collected on an empty stomach the second day after admission. The serum Hcy level was measured within 24 h of hospitalization with the enzymatic cycling method. HHcy was defined as Hcy concentration ≥12.0 *μ*mol/L [10]. Full neurological examination, brain CT or MRI scan, and carotid ultrasonography were also recorded.

### 2.3. Brain MRI Acquisition and Analysis

The MR examination was performed within 48 hours after admission, and sequences included T1-weighted, T2-weighted, fluid-attenuated inversion recovery (FLAIR), axial diffusion-weighted imaging (DWI), and TOF-MRA sequences. MR was rated for the presence of lacunes, WMHs, CMBs, and PVS independently. Lacunes were defined as rounded or ovoid-shaped lesions, >3- and <20 mm diameter on T1, T2, or FLAIR sequences in the basal ganglia, white matter, or brainstem. We defined CMBs as small (<5 mm), homogeneous, round foci of low signal intensity on gradient echo images of the basal ganglia, white matter, cerebellum, brainstem, or corticosubcortical junction [11]. PVS was defined as small, (<3 mm) round, or linear hyperintensities in the basal ganglia or centrum semiovale on T2 images, and they were rated using a five-point ordinal scale12 as follows: 0 = no PVS, 1 = 1–10 PVS, 2 = 11–20, PVS, 3 = 21–40 PVS, and 4 = >40 PVS. Three trained neurologists and 2 neuroradiologists, each of whom was blinded to clinical information rated all the available scans for the presence and severity of SVD features. Deep and periventricular WMHs were both coded according to the Fazekas scale from 0 to 3 [12].

Based on the recent described score [4], we counted the presence of each SVD feature and rated the total burden of SVD on an ordinal scale from 0 to 4. The MR manifestation of WMHs graded 2-3 according to the Fazekas grading was recorded as 1 point, presence of CMBs or lacunes was recorded as 1 point, respectively, and PVS graded 2–4 (≧11) was counted 1 point (Table 1). The severity of the total SVD burden score was divided into three categories: mild (0 or 1 point), moderate (2 points), and severe (3 or 4 points) [3, 13].

### 2.4. Statistical Analysis

Statistical analysis was performed with SPSS13.0 (SPSS, Inc., Chicago, IL, USA). Normally distributed variables were presented as mean ± Standard Deviation (SD), and categorical data were presented as frequency or ratio. Kolmogorov–Smirnov test was used to test normality of quantitative data. Levene's test was used to test homogeneity of variance. One-way ANOVA was performed to compare the distribution of quantitative variables. The *χ*^2^ test was used to compare the distribution of classification index. To determine the independent factors related to CSVD, we performed one variable analysis and multiple logistic regression analysis by using a backward elimination method and set the probability at 0.10 for removal. The statistical significance level was set at *P* < 0.05 in the present study.

## 3. Results

### 3.1. Baseline Characteristics

A total of 329 (220 males and 109 females) participants were finally included, and they were divided into four groups. There were 76 participants (23.10%) in the control group, 112 participants (34.04%) in the isolated HBP group, 53 participants (16.11%) in the isolated HHcy group, and 88 participants (26.75%) in the H-type hypertension group at baseline.  Table 2 summarized the clinical characteristics of patients with H-type hypertension. Participants with H-type hypertension were more likely to be elderly and male, have higher systolic pressure, have a higher level of uric acid and Hcy, and have a medical history of diabetes mellitus than the control participants. When comparing the isolated hypertension group with the H-type hypertension group, higher Hs-CRP (4.24 ± 4.69 vs 6.12 ± 6.77) level was observed in H-type hypertension participants (*P* < 0.05), as well as gender and uric acid (283.55 ± 78.97 vs 344.58 ± 106.09) and Hcy level (8.81 ± 2.49 vs 22.46 ± 27.68) had a significant difference between the two groups (*P* < 0.01). Diabetes (1.9% vs 22.7%) and systolic pressure (134.39 ± 14.42 vs 147.19 ± 19.24) had a significant difference between the isolated HHcy group and the H-type hypertension group (all *P* < 0.01).

### 3.2. Correlation between H-Type Hypertension and CSVD Spectrum

**Figure 1:**
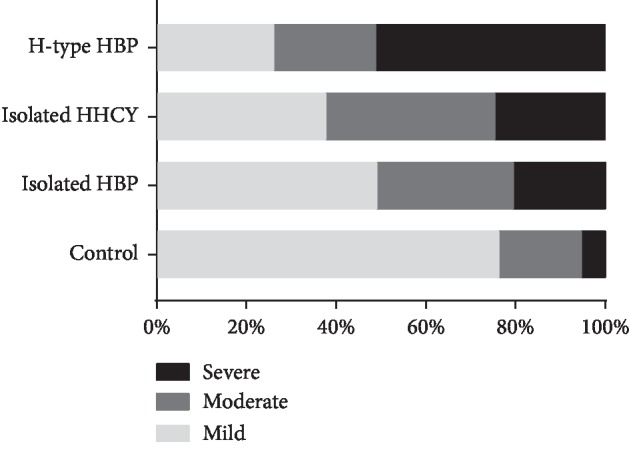
The comparison of CSVD total burden among the four groups. Note: Mild CSVD total burden: 0 or 1 point, moderate CSVD total burden: 2 points, severe CSVD total burden:3 or 4 points.

CSVD spectrum included WMHs, LI, CMBs, and PVS. We analysed the correlation between H-type hypertension and these subtypes, and our results showed that the H-type hypertension participants were more likely susceptible to the presence of CMBs, more serious WMHs and PVS, and higher CSVD burden score than the control group. When it came to the H-type hypertension group and the isolated HBP group, more serious WMHs, higher frequency of CMBs occurrence, and higher CSVD total burden score could be observed in the H-type hypertension participants. More patients suffered from moderate-to-severe WMHs and CMBs presence in the H-type hypertension group compared with the isolated HHcy group (Table 3 and Figure 1).

### 3.3. The Association of Risk Factors with CSVD Total Burden

A number of predictors of CSVD were shown in the logistic regression model (Table 4). Single factor analysis indicated that age, systolic pressure, and diastolic pressure were contributed to the high CSVD total burden. However, after the multivariate adjustment, CSVD total burden was significantly related to age (OR: 1.059, 95% CI: 1.037–1.082), systolic pressure (OR: 1.122, 95% CI: 1.007–1.136), and triglycerides (OR: 1.386, 95% CI: 1.037–1.854).

### 3.4. The Association of H-Type Hypertension with CSVD Total Burden

In univariate analysis, baseline-isolated hypertension (OR: 3.339, 95% CI: 1.751–6.369), isolated HHcy (OR: 5.317, 95% CI: 2.469–11.447), and H-type hypertension (OR: 9.667, 95% CI: 4.725–19.778) were all associated with the CSVD total burden. In multivariate analysis, we adjusted for age, systolic pressure, and triglycerides and found that isolated HHcy (multivariate-adjusted OR: 4.154, 95% CI: 1.836–9.401) and H-type hypertension (multivariate-adjusted OR: 5.028, 95% CI: 2.323–10.883) were indicators of CSVD total burden. Finally, we investigated whether there was synergistic association between hypertension and HHcy. The results showed that, after the multivariate adjustment of age, systolic pressure, triglycerides, hypertension, and HHcy had a synergistic effect on CSVD total burden (OR: 2.776, 95% CI: 1.564–4.927). The results are shown in Table 4.

## 4. Discussion

A direct relationship between Hcy and CSVD spectrum has been observed in several studies. One study reported that patients who suffered from CSVD with confluent leukoaraiosis had the highest serum Hcy level compared with other TOAST subtypes of stroke [14]. Another study found that, in Japanese patients, elevated Hcy level is independently associated with leukoaraiosis, but not with the incidence of microbleeds [15]. Lin et al. reported both smoking and total HCY levels were shown to be risk factors for lacunes occurrence [16]. Wang et al. observed an independent association between Hcy level and severity of the CMBs [17]. Miwa et al. reported that serum Hcy was associated with lacunas, CMBs, and strictly deep CMBs [18]. The pathologic progress related to cerebral microvascular diseases include lots of pathophysiological pathways, one of which is endothelial dysfunction or even direct neurotoxicity [19]. HCY is known to have a direct toxic effect on the endothelium [19]. Another article reported that elevated Hcy level increases hypercogulability [20] and oxidative stress [3] induces endothelial dysfunction and smooth muscle cell proliferation [21], increases hypercoagulability, and thus contributed to the damage of blood brain barrier.

Our study is the first to focus on the predictive impact of H-type hypertension on CSVD. In our research population, we studied the relationship between H-type hypertension and CSVD spectrum and observed that H-type hypertension was associated with the presence of CMBs and the severity of WMHs and PVS.

However, CSVD imaging features usually occur together. A relative study showed that signs of two or more severe CSVD features may appear in around one-third of the patients suffering from acute ischemic stroke. A number of researches indicated that quantification of the global burden of CSVD is feasible, meaningful, and has clinical relevance [22, 23]. The total CSVD burden may provide a more comprehensive view of the global impact of CSVD than the single feature separately. Thus, in the present study, we aimed to study the effect of H-type hypertension on the CSVD total burden. We observed that isolated homocysteine and H-type hypertension were associated with CSVD total burden. Like other traditional risk factors, such as age, systolic pressure, and triglycerides, H-type hypertension is an independent risk factor for CSVD total burden. Our study is the first to focus on the predictive impact of H-type hypertension on CSVD spectrum and CSVD total burden.

In the present study, CSVD total burden was significantly related to age, systolic pressure, triglycerides, isolated HHcy, and H-type hypertension. Also, we further observed hypertension and HHcy had a synergistic effect on CSVD total burden (OR: 2.776, 95% CI: 1.564–4.927).

A certain synergistic effect of hypertension and HHcy can be observed in some recent studies [24, 25]. A large population-based study showed that H-type hypertension contributed to a remarkable increase of stroke incidence compared with isolated HBP [26]. Another study in which they enrolled 750 subjects of cardiac, cerebral, and peripheral reported disease reported that the incidence of atherosclerotic vascular diseases was about five times higher than that of the patients who suffered from the isolated hypertension [27]. The primary mechanisms may be explained by the fact that HHcy activates the angiotensin-converting enzyme by inhibiting the production of endogenous hydrogen sulfide to aggravate hypertension [28–30]. Another study discovered that reduction of Treg cells percentage might be an important cause of immune disorders in H-type hypertension patients. According to their results, HHcy oxidized to peroxide, causing T-cell subsets imbalance and vascular injury aggravation [10]. Guo et al. found that the risk for plaque occurrence in patients with H-type hypertension was 1.63 times of patients with simple (or isolated) systolic hypertension. They further discovered that high homocysteine concentration might aggravate the oxidative stress in hypertension to produce contributory effects on vascular impairment [31]. Thus, when hypertension and HHcy are combined, the adverse effects may be increased.

The potential limitations of the study need to be acknowledged. First, vitamin B12, pyridoxal-5-phosphate, and some medicines are related to Hcy metabolism and may affect the results. However, in our study, we only tested the plasma homocysteine level and did not record patients' medications at baseline. In future follow-up studies, we will add these parameters and expect to further interpret the relationship between H-type hypertension and CSVD. Subsequently, we only detected blood pressure and the level of plasma Hcy at one time point and have no data on possible changes in the long term. Finally, the sample size in our study was relatively small that might have an impact on the overall assessment of the results.

In conclusion, our study indicated that H-type hypertension was associated with CSVD total burden and CSVD spectrum, which deserves further prevention measures. Furthermore, hypertension and HHcy had a synergistic effect on CSVD total burden.

## Figures and Tables

**Table 1:** The scale of CSVD total burden.

CSVD MRI spectrum	White matter hyperintensities	Perivascular space	Microbleeds	Lacunes
Visual assessment	Fazekas scale	Semiquantitative scale	Consensus definition	Consensus definition
Grade	Perivebtricular WMH Fazekas 3 and/or deep WMH Fazekas 2-3	Semiquantitative scale 2–4	≥1 microbleed	≥1 lacune
Score	1 point	1 point	1 point	1 point

**Table 2:** Clinical baseline characteristics of participants according to H-type hypertension.

Variables	Control group (*n* = 76)	Isolated HBP (*n* = 112)	Isolated HHcy (*n* = 53)	H-type HBP group (*n* = 88)	*χ* ^2^/*F*	*P*
Age	55.78 ± 13.36	63.35 ± 12.82^*∗∗*^	63.45 ± 13.03^*∗*^	66.44 ± 13.15^*∗∗*^	9.624	0.000
Male	49 (64.5%)	65 (58.0%)	36 (67.9%)	70 (79.5%)^*∗*##^	10.551	0.014
Diabetes mellitus	7 (9.3%)	32 (28.6%)^*∗∗*^	1 (1.9%)^##^	20 (22.7%)^*∗*ΔΔ^	22.647	0.000
Cardiac diseases	12 (15.8%)	16 (14.3%)	11 (21.2%)	19 (21.6%)	2.429	0.488
Systolic pressure	132.33 ± 13.04	148.80 ± 17.86^*∗∗*^	134.39 ± 14.42^##^	147.19 ± 19.24^*∗∗*ΔΔ^	21.022	0.000
Diastolic pressure	79.08 ± 9.69	85.78 ± 11.75^*∗∗*^	79.66 ± 10.74^##^	82.98 ± 14.48	5.900	0.001
TC (mmol/l)	4.11 ± 0.92	4.28 ± 1.08	3.99 ± 0.83	4.39 ± 1.02	2.274	0.080
TG (mmol/l)	1.34 ± 0.60	1.72 ± 1.28	1.17 ± 0.46^##^	1.64 ± 1.19	4.453	0.004
HDL-C (mmol/l)	1.19 ± 0.28	1.25 ± 0.34	1.17 ± 0.30	1.21 ± 0.29	1.222	0.302
LDL-C (mmol/l)	2.38 ± 0.80	2.54 ± 0.89	2.31 ± 0.70	2.63 ± 0.75	2.347	0.073
Glucose (mmol/l)	5.80 ± 2.28	6.90 ± 2.70^*∗*^	5.24 ± 0.99^##^	6.27 ± 1.91	7.876	0.000
Urid acid (U/L)	281.40 ± 74.02	283.55 ± 78.97	313.71 ± 90.11	344.58 ± 106.09^*∗∗*##^	10.187	0.000
Hs-CRP (mg/L)	4.19 ± 6.27	4.24 ± 4.69	4.18 ± 4.65	6.12 ± 6.77^#^	2.441	0.064
Hcy (*μ*mol/l)	8.00 ± 2.29	8.81 ± 2.49	22.60 ± 20.86^*∗∗*#^	22.46 ± 27.68^*∗∗*##^	19.049	0.000

Note: ^*∗∗*^*P* < 0.01, ^*∗*^*P* < 0.05 vs. control group, ^##^*P* < 0.01, ^#^*P* < 0.05 vs. isolated hypertension group, ^ΔΔ^*P* < 0.001, ^Δ^*P* < 0.005 vs. isolated HHcy group. HBP is the abbreviation of hypertension.

**Table 3:** Correlation between H-type hypertension and CSVD spectrum.

CSVD spectrum	Control group	Isolated HBP	Isolated HHcy	H-type HBP group	*χ* ^2^/*F*	*P*
Moderate-to-severe WMH	9 (11.8%)	36 (32.1%)^*∗∗*^	16 (30.2%)^*∗*^	55 (62.5%)^*∗∗*##ΔΔ^	47.938	0.000
LI presence	41 (53.9%)	78 (69.6%)^*∗∗*^	34 (64.2%)	56 (63.6%)	4.827	0.185
CMBs presence	6 (7.9%)	21 (18.8%)	16 (30.2%)^*∗*^	45 (51.1%)^*∗∗*##Δ^	44.478	0.000
PVS grade 2–4	17 (22.4%)	39 (34.8%)	25 (47.2%)^*∗*^	51 (58.0%)^*∗*^	14.696	0.002
Moderate-to-severe CSVD burden score	18 (23.7%)	57 (50.9%)^*∗∗*^	33 (62.3%)^*∗∗*^	66 (75%)^*∗∗*#^	45.331	0.000

Note: ^*∗∗*^*P* < 0.01, ^*∗*^*P* < 0.05 vs. control group, ^##^*P* < 0.01, ^#^*P* < 0.05 vs. isolated hypertension group, ^ΔΔ^*P* < 0.001, ^Δ^*P* < 0.005 vs. isolated HHcy group. HBP is the abbreviation of hypertension.

**Table 4:** The association of risk factors with CSVD total burden.

Risk factors	Single factor analysis		Multiple analysis	
	OR (95%CI)	*P*	OR (95%CI)	*P*
Age	1.048 (1.029–1.067)	0.000	1.059 (1.037–1.082)	0.000
Gender	0.913 (0.577–1.446)	0.073	0.632 (0.373–1.071)	0.088
Diabetes mellitus	1.413 (0.800–2.497)	0.234	1.387 (0.694–2.770)	0.588
Cardiac diseases	1.456 (0.819–2.594)	0.203	1.068 (0.544–2.096)	0.345
Systolic pressure	1.026 (1.013–1.040)	0.000	1.122 (1.007–1.136)	0.006
Diastolic pressure	1.020 (1.001–1.039)	0.037	1.014 (0.989–1.041)	0.276
Total cholesterol	1.018 (0.819–1.266)	0.871	0.823 (0.631–1.073)	0.062
Triglycerides	1.200 (0.948–1.518)	0.129	1.386 (1.037–1.854)	0.015
LDL	1.046 (0.800–1.368)	0.742	1.123 (0.549–2.296)	0.549
HDL	0.798 (0.394–1.617)	0.531	0.657 (0.251–1.720)	0.854
Glucose	1.040 (0.944–1.145)	0.430	0.957 (0.854–1.07)	0.456
Uric acid	1.001 (0.999–1.004)	0.225	1.001 (0.998–1.004)	0.521
Hs-CRP	1.020 (0.982–1.061)	0.305	1.010 (0.964–1.059)	0.980
Control	1.000		1.000	
Isolated hypertension	3.339 (1.751–6.369)	0.000	1.611 (0.779–3.333)	0.198
Isolated HHcy	5.317 (2.469–11.447)	0.011	4.154 (1.836–9.401)	0.001
H-type hypertension	9.667 (4.725–19.778)	0.022	5.028 (2.323–10.883)	0.007
HBP by Hcy	3.694 (2.142–6.373)	0.009	2.776 (1.564–4.927)	0.002

## Data Availability

The data used to support the findings of this study are available from the corresponding author upon request.
